# Cross-Linked Metathesis Polynorbornenes Based on Nadimides Bearing Hydrocarbon Substituents: Synthesis and Physicochemical Properties

**DOI:** 10.3390/polym16182671

**Published:** 2024-09-22

**Authors:** Kirill S. Sadovnikov, Ivan V. Nazarov, Vsevolod A. Zhigarev, Anastasia A. Danshina, Igor S. Makarov, Maxim V. Bermeshev

**Affiliations:** 1 A.V. Topchiev Institute of Petrochemical Synthesis, Russian Academy of Sciences, 29 Leninskiy Prospekt, 119991 Moscow, Russia; k.sadovnikov@ips.ac.ru (K.S.S.); vanyanaz@ya.ru (I.V.N.); zhigarev@ips.ac.ru (V.A.Z.); makarov@ips.ac.ru (I.S.M.); 2 A.N. Nesmeyanov Institute of Organoelement Compounds, Russian Academy of Sciences, 28 Vavilova Str., 119334 Moscow, Russia; danshina.aa@phystech.edu; 3 Moscow Center for Advanced Studies, 20, Kulakova Str., 123592 Moscow, Russia

**Keywords:** nadimide, metathesis polymerization, polynorbornene, β-pinene, isophoronediamine

## Abstract

Metathesis homo- and copolymerization of bifunctional monomers bearing two norbornene moieties was studied. The monomers were synthesized from cis-5-norbornene-exo-2,3-dicarboxylic anhydride and various diamines (hexamethylenediamine, decamethylenediamine, 1R,3S-isophoronediamine). The metathesis homopolymerization of these bis(nadimides) in the presence of the second-generation Grubbs catalyst afforded glassy cross-linked polymers in more than 90% yields. The metathesis copolymerization of the bis(nadimides) and a monofunctional norbornene derivative containing the β-pinene fragment also resulted in insoluble cross-linked polymers in nearly quantitative yields. The structures and purity of the synthesized polymers were confirmed via IR spectroscopy and CP/MAS NMR spectroscopy. Conditions for the fabrication of mechanically strong solution-cast thin films based on copolymers synthesized from the comonomers mentioned above were determined by varying the content of the cross-linking agent. It was shown that the films made in this way are stable in a range of organic solvents and could be useful as semipermeable or membrane materials for use in liquid organic media. The permeability of the polymer films in question to 1-phenylethanol and mandelic acid was studied. The results obtained are discussed along with the data from the DSC, TGA, and powder X-ray diffraction studies of the properties of the synthesized metathesis homo- and copolymers.

## 1. Introduction

Metathesis polymers based on norbornenes are convenient objects for systematic studies and the establishment of structure–property correlations. This is first of all due to the availability of the starting monomers [1,2,3,4] necessary to synthesize the corresponding polymers and the high reactivity of norbornene derivatives in the metathesis reaction [5,6,7]. Additionally, highly effective single-component catalysts for ring-opening metathesis polymerization (ROMP) were developed, e.g., the Schrock and Grubbs catalysts [8,9,10,11,12]. They exhibit high activity and are tolerant of the functional groups present in a monomer, which allows one to significantly reduce the substituent effect on the polymerization process and enables facile synthesis of polymers with desired substituents and specified molecular weights. Currently, metathesis polymers based on unsubstituted norbornene, as well as hydrogenated metathesis polymers based on norbornenes bearing hydrocarbon substituents, are produced industrially under different trade names [13] and used as vibration-absorbing materials, sorbents for the collection of oil and petroleum spills, transparent materials for optoelectronics, etc.

Through the functionalization of the side chains of metathesis polymers, one can enhance or even add new properties to the polymers. For instance, the introduction of ethylene glycol or ester groups makes it possible to obtain amphiphilic polymers [14,15,16,17], the presence of bulky organosilicon or carbocyclic groups considerably increases gas permeability [18,19,20], modification with polar groups enhances the separation selectivity of CO_2_-containing gas mixtures [21,22,23,24], and organofluorine groups cause the dielectric constant of polymers to decrease [25,26]. A simple and versatile platform for the synthesis of various metathesis polymers is provided by exo-norbornenedicarboxylic acid anhydride, which can be obtained from readily available industrial products such as dicyclopentadiene and maleic anhydride [27]. The presence of a reactive anhydride fragment in this molecule offers prospects for the synthesis of various norbornene derivatives bearing ester, amido, or imido groups [28,29,30]. Earlier, it was demonstrated that the metathesis and addition polymers based on norbornenes bearing imido groups (nadimides) are of great interest as membrane materials for the separation of gases [31,32,33,34,35,36,37], organic compounds and their enantiomers, and other applications [38,39].

The polymers in question often exhibit excellent performance characteristics; however, they should also meet a key requirement of being stable in various environments, e.g., organic solvents. This can be achieved by cross-linking, which dramatically decreases the solubility and swelling of polymers. One way to make cross-linked polynorbornenes is to copolymerize a norbornene derivative with another derivative that has two reactive norbornene moieties. In this work, we carried out detailed studies of the metathesis homopolymerization of three bifunctional monomers synthesized from cis-5-norbornene-exo-2,3-dicarboxylic anhydride and different diamines, as well as the metathesis copolymerization of these three monomers and a related derivative containing a bulky pinanyl group. Having optimized the polymerization conditions, we obtained cross-linked polymers, fabricated films characterized by good mechanical properties and stability in organic solvents, and studied the permeability of the films to 1-phenylethanol and mandelic acid in various organic media.

## 2. Materials and Methods

### 2.1. Materials

The second-generation Grubbs catalyst (purity 97%, Merck, Union County, NJ, USA, product No. 73304), ethyl vinyl ether (purity 99%, stabilized with 0.1% KOH, Merck, product No. 422177), *cis*-5-norbornene-*exo*-2,3-dicarboxylic anhydride (**NDA**) (purity > 97%, TCI, Tokyo, Japan, product No. 0767), (-)-*β*-pinene (purity > 94%, TCI, product No. 18172-67-3), borane dimethyl sulfide complex (purity > 98%, Merck, product No. 179825-800ML), hydroxylamine-*O*-sulfonic acid (purity > 98%, Merck, product No. 213136), 1,6-diaminohexane (purity > 98%, Merck, product No. H11696), 1,10-diaminodecane (purity > 97%, Merck, product No. D14204), isophoronediamine (purity > 99%, Merck, product No. 118184), benzoic acid (purity > 99.5%, Merck, product No. W213101), 1-phenylethanol (purity 98%, Merck, product No. P13800), and mandelic acid (purity 99%, Merck, product No. M2101) were used without additional purification. (-)-*cis*-Myrtanylamine [40] and pinanyl-substituted nadimide (**NBpin**) [41] were synthesized following published procedures. (1*R*,3*S*)-Isophoronediamine was isolated as reported elsewhere [42]. Toluene and THF were dehydrated using an appropriately equipped M-Braun SPS-7 solvent purification system. Chloroform (“chemically pure” grade, TD Chimmed, Moscow, Russia) and deuterated chloroform (purity 99.9%, stabilized with Ag, Solvex, Moscow, Russia) were refluxed over CaH_2_ (96%, TD Chimmed, Moscow, Russia) for 3 h and distilled in argon atmosphere (purity 99.998%, Argon, Moscow, Russia). Methanol (“chemically pure” grade, TD Chimmed, Moscow, Russia) was refluxed over magnesium (“chemically pure” grade, Mosreaktiv, Moscow, Russia) for 3 h and distilled in argon atmosphere (purity 99.998%, Argon, Moscow, Russia).

### 2.2. Methods for Characterization of Monomers and Polymers Obtained via Metathesis Polymerization

Nuclear magnetic resonance (NMR) spectra were recorded on a Bruker (Billerica, MA, USA) AvanceTM DRX400 spectrometer operating at 400.1 MHz (^1^H) and 100.6 MHz (^13^C) in CDCl_3_ (purity 99.9%, stabilized with Ag, Solvex, Moscow, Russia). Signals in the ^1^H and ^13^C NMR spectra were assigned using the residual proton signal of CDCl_3_ (δ 7.26) and the central peak of CDCl_3_ (δ 77.00) as the corresponding references.

High-resolution ^13^C NMR spectra of solid samples were recorded on a Varian Unity Inova AS500 (Agilent Technologies, Santa Clara, CA, USA) instrument operating at 125.5 MHz using the cross-polarization magic angle spinning (CP/MAS) technique. The MAS probe rotor had an outer diameter of 3.2 mm and was spun at 15 kHz. The duration of the 90° pulse, that of the contact pulse, and the time delay was set to 3.5 µs, 2 ms, and 2 s, respectively. The ^13^C chemical shift axis was calibrated using the signal from the carbonyl C atom in glycine (δ 176.03) as the secondary reference.

A gas chromatography/mass spectrometry analysis was performed using a Finnigan MAT 95 XL mass spectrometer coupled to an Agilent HP 6890+ chromatograph (Agilent Technologies, Santa Clara, CA, USA). The operating parameters of the mass spectrometer were as follows: ionization energy 70 eV, mass scan range 20–800 amu, resolution 1000, source temperature 200 °C, and a mass scan rate of 1 s per mass decade. The chromatographic conditions were a 30 m × 0.25 mm capillary column packed with the DB-5 stationary phase (95% polydimethylsiloxane, 5% phenyl groups), He as the carrier gas (purity 99.995%, NII KM, Moscow, Russia), flow split ratio 1:30, heating from 30 to 120 °C at a rate of 5 deg Celsius min^−1^, heating from 120 to 270 °C at a rate of 10 deg Celsius min^−1^, and subsequent maintaining at 270 °C for 10 min.

IR spectra were recorded on a Bruker IFS-66 v/S FT-IR spectrometer (Bruker, Ettlingen, Germany) operating in the attenuated total reflectance mode. The experimental parameters were as follows: a ZnSe crystal, spectral range 4000–600 cm^−1^, resolution 2 cm^−1^, and 15 scans.

X-ray diffraction data for **NBI** (the crystals of **NBI** were obtained via the slow evaporation of the solution of **NBI** in the mixture of CH_2_Cl_2_ and CH_3_CN (2/1 *v*/*v*)) were collected at 120 K with a Bruker APEX2 DUO CCD diffractometer, using graphite monochromatized Mo-Kα radiation (* = 0.71073 Å, ω-scans). The structure was solved using intrinsic phasing with the ShelXT [43] structure solution program in Olex2 (version – 1.5) [44] and refined with the XL [45] refinement package using least-squares minimization against F2 in the anisotropic approximation for non-hydrogen atoms. Positions of hydrogen atoms were calculated and then refined in the isotropic approximation within the riding model. Crystal data and structure refinement parameters are given in Table S1. CCDC 2370134 contains the supplementary crystallographic information for this paper.

Calorimetric measurements were carried out on a Mettler TA-4000 (Giesen, Germany) differential scanning calorimeter equipped with a DSC-30 heating cell in argon atmosphere at a heating rate of 20 deg Celsius min^−1^. Thermogravimetric analysis (TGA) was performed using a “TGA/DSC 1” (Mettler Toledo, Polaris Parkway, OH, USA) in argon and in air at the heating rate of 10 °C/min from 30 to 1000 °C.

Powder X-ray diffraction (XRD) data were collected on an Empyrean (Malvern Panalytical, Almelo, The Netherlands) equipped with a Bruker AXS detector (CuKα radiation, λ = 1.54 Å). Distances between structural fragments were calculated using the Wulff–Bragg’s condition.

The specific rotation of the monomers and polymers was measured using a KRÜSS P3000 polarimeter (A.KRÜSS Optronic GmbH, Hamburg, Germany) in CHCl_3_ (“for HPLC” grade, purity > 99.8%, Merck, product No. 34854).

Density was measured via hydrostatic weighing using water. A film sample was weighed on an analytical balance (m_dry_). A vessel was filled with methanol and placed on the balance pan. A thin copper wire was suspended above the balance. The sample was attached to the lower end of the wire, immersed in methanol, and weighed (m_1_). Then, the sample was removed and the pendant wire immersed in methanol was weighed (m_2_). The density of the film sample was calculated using the expression d = d_s_ m_dry_/(m_dry_ − (m_1_ − m_2_)), where d_s_ = 0.791 g mL^−1^ is the density of methanol.

Mechanical tests of the films were carried out on an I1140M-5-01-1 universal tensile testing machine (Tochpribor-KB, Ivanovo, Russia) using the ASTM D638 method. Dog-bone specimens (ASTM standard D1708-96 [46], 22 × 5 mm) were prepared by punching the films using a stainless steel die. The data are presented as the mean ± standard deviation (SD) of mean and as the median with interquartile range. One-way ANOVA followed by Dunnett’s test was performed using the Sigma Stat 3.5 (Systat Software, San José, CA, USA). The *p* values meeting the condition *p* < 0.05 were considered statistically significant.

An MBraun LABstar (Stratham, NH, USA) glovebox was used for manipulations in inert atmosphere.

### 2.3. Synthetic Part

#### 2.3.1. Synthesis of Monomers

**General procedure 1.** To a suspension of **NDA** (10 mmol) in toluene (3 mL), diamine (5 mmol) in toluene (1 mL) was added at 40 °C. After cooling to room temperature and stirring for one hour, the mixture was heated to 110 °C and refluxed for two hours. The solvent was removed at a reduced pressure and the product was purified via recrystallization from a CHCl_3_/CH_3_OH mixture or from CH_3_OH.

#### 2.3.2. 2,2′-Hexane-1,6-diylbis(3a,4,7,7a-tetrahydro-1H-4,7-methanoisoindole-1,3-dione) (NB6) [47]

White crystals; yield 92% (1877 mg); T_m_ = 151–153 °C. ^1^H NMR spectrum (400 MHz, CDCl_3_): *δ* 1.15–1.24 (m, 2H, CH_2_), 1.25–1.36 (m, 4H, 2CH_2_), 1.44–1.59 (m, 6H, 3CH_2_), 2.66 (s, 4H, 2CH_2_), 3.26 (s, 4H, 2CH_2_), 3.42 (t, 4H, ^3^*J* = 7.40 Hz, 2CH_2_), 6.27 (s, 4H, 4CH=). ^13^C NMR spectrum (100 MHz, CDCl_3_): *δ* 26.6 (2CH_2_), 27.7 (2CH_2_), 38.6 (2CH_2_), 42.8 (2CH_2_), 45.2 (4CH), 47.9 (4CH), 137.9 (4CH=), 178.2 (4C(O)N).

#### 2.3.3. 2,2′-Decane-1,10-diylbis(3a,4,7,7a-tetrahydro-1H-4,7-methanoisoindole-1,3-dione) (NB10) [48]

White crystals; yield 88% (2042 mg); T_m_ = 93–95 °C. ^1^H NMR spectrum (400 MHz, CDCl_3_): *δ* 1.17–1.34 (m, 14H, CH_2_), 1.46–1.59 (m, 6H, 3CH_2_), 2.66 (s, 4H, 2CH_2_), 3.27 (s, 4H, 2CH_2_), 3.44 (t, 4H, ^3^*J* = 7.62 Hz, 2CH_2_), 6.28 (s, 4H, 4CH=). ^13^C NMR spectrum (100 MHz, CDCl_3_): *δ* 27.0 (2CH_2_), 27.8 (2CH_2_), 29.2 (2CH_2_), 29.4 (2CH_2_), 38.8 (2CH_2_), 42.8 (2CH_2_), 45.3 (4CH), 47.9 (4CH), 137.9 (4CH=), 178.2 (4C(O)N).

#### 2.3.4. 2-((1R,3S)-3-[(1,3-Dioxohexahydro-1H-4,7-methanoisoindole-2-yl)methyl]-3,5,5-trimethylcyclohexyl)hexahydro-1H-4,7-methanoisoindole-1,3-dione (NBI)

White crystals; yield: 59% (1363 mg); T_m_ = 189–190 °C. ^1^H NMR spectrum (400 MHz, CDCl_3_): *δ* 0.93 (s, 3H, CH_3_), 1.04 (s, 3H, CH_3_), 1.07 (s, 3H, CH_3_), 1.16–1.36 (m, 6H, 3CH_2_), 1.44–1.60 (m, 2H, CH_2_), 1.95–2.16 (m, 2H, CH_2_), 2.58 (s, 2H, CH_2_), 2.64–2.74 (m, 2H, CH_2_), 3.18–3.33 (m, 6H, 3CH_2_), 4.30 (tt, 1H, ^3^*J* = 12.84 Hz, ^4^*J* = 3.30 Hz, 2CH_2_), 6.23–6.32 (m, 4H, 4CH=). ^13^C NMR spectrum (100 MHz, CDCl_3_): *δ* 23.7, 27.5, 32.0, 35.2, 37.2, 38.4, 40.5, 42.7, 43.1, 45.3, 45.4, 45.48, 45.50, 45.7, 47.2, 47.4, 47.8, 51.7, 137.82 (CH=), 137.94 (CH=), 138.0 (2CH=), 178.22 (C(O)N), 178.32 (C(O)N), 178.7 (2C(O)N).

MS (ESI) *m*/*z* for C_28_H_35_N_2_O_4_^+^ [M + H]^+^: 463.2.

#### 2.3.5. Synthesis of Cross-Linked Homopolymers

**General procedure 2.** To a diimide solution (0.2 mmol, 500 eq.) in THF (2 mL), the second-generation Grubbs catalyst (1.7 mg, 4 × 10^−4^ mmol, 1 eq.) in THF (0.5 mL) was added in an inert atmosphere, and the mixture was stirred at 20 °C for 24 h. Then, ethyl vinyl ether (0.2 mL) was added to the reaction mixture to complete the polymerization reaction. The polymer thus obtained was purified with methanol using a Soxlet apparatus.

#### 2.3.6. Homopolymer Based on **NBI**

White powder; yield 91% (85.4 mg).

^13^C CP/MAS NMR spectrum: *δ* 15.3–63.7 (m, C, CH, CH_2_, CH_3_), 120.4–147.1 (m, CH=), 172.3–185.9 (m, C(O)N).

IR (ATR, cm^−1^): ν(C−H) 3079−2782 (m), ν(C=O_imido_) 1772 (s), ν(C=O_imido_) 1692 (s), ν(CH=CH_trans_) 966 (s).

#### 2.3.7. Homopolymer Based on **NB6**

White powder; yield 91% (74.3 mg).

^13^C CP/MAS NMR spectrum: *δ* 14.3−69.3 (m, C, CH, CH_2_, CH_3_), 113.7−143.2 (m, CH=), 169.9−184.1 (m, C(O)N).

IR (ATR, cm^−1^): ν(C−H) 3062−2792 (m), ν(C=O_imido_) 1768 (s), ν(C=O_imido_) 1689 (s), ν(CH=CH_trans_) 968 (s).

#### 2.3.8. Homopolymer Based on **NB10**

White powder; yield 94% (87.3 mg).

^13^C CP/MAS NMR spectrum: *δ* 11.3−58.9 (m, C, CH, CH_2_, CH_3_), 118.1−153.3 (m, CH=), 167.4−184.4 (m, C(O)N).

IR (ATR, cm^−1^): ν(C−H) 3069−2782 (m), ν(C=O_imido_) 1772 (s), ν(C=O_imido_) 1692 (s), ν(CH=CH_trans_) 968 (s).

### 2.4. Synthesis of Membranes Based on Cross-Linked Copolymers

**General procedure 3** (exemplified by the synthesis of the copolymer of **NBpin** (90 mol.%) and **NBI** (10 mol.%)).

To a mixture of **NBpin** (360 mg, 1.2 mmol) and **NBI** (40 mg, 8.6 × 10^−2^ mmol) in CHCl_3_ (6 mL)—a total of 500 eq.—, the second-generation Grubbs catalyst (4.4 mg, 5.2 × 10^−3^ mmol, 1 eq.) in CHCl_3_ (4 mL) was added at 25 °C. The reaction mixture was thoroughly stirred and the solution thus obtained was filtered and cast to fully cover the area within a metallic ring 7 cm in diameter placed on a cellophane substrate. The metallic ring with the filtered reaction mass was covered with a Petri dish, and the solvent was allowed to evaporate for 24 h. The fabricated membrane was immersed in methanol for 24 h and then dried in vacuo. The thickness of the polymer film varied in the range of 77–97 µm.

*Copolymer of **NBpin** and **NBI** (90:10 mol.%)*

IR (ATR, cm^−1^): ν(C−H) 3064−2757 (m), ν(C=O_imido_) 1772 (s), ν(C=O_imido_) 1694 (s), ν(CH=CH_trans_) 967 (s), ν(CH=CH_cis_) 761 (s).

*Copolymer of **NBpin** and **NBI** (50:50 mol.%)*

^13^C CP/MAS NMR spectrum: *δ* 16.9−67.2 (m, C, CH, CH_2_, CH_3_), 124.3−145.5 (m, CH=), 175.6−188.1 (m, C(O)N).

*Copolymer of **NBpin** and **NBI** (40:60 mol.%)*

^13^C CP/MAS NMR spectrum: *δ* 15.6−64.7 (m, C, CH, CH_2_, CH_3_), 118.2−148.7 (m, CH=), 176.6−188.9 (m, C(O)N).

*Copolymer of **NBpin** and **NB6** (90:10 mol.%)*

IR (ATR, cm^−1^): ν(C−H) 3062−2775 (m), ν(C=O_imido_) 1771 (s), ν(C=O_imido_) 1693 (s), ν(CH=CH_trans_) 967 (s), ν(CH=CH_cis_) 761 (s).

*Copolymer of **NBpin** and **NB10** (90:10)*

IR (ATR, cm^−1^): ν(C−H) 3062−2780 (m), ν(C=O_imido_) 1771 (s), ν(C=O_imido_) 1693 (s), ν(CH=CH_trans_) 967 (s), ν(CH=CH_cis_) 760 (s).

### 2.5. Permeation Study of 1-Phenylethanol and Mandelic Acid through Membranes Based on the Synthesized Metathesis Copolymers

**Figure 1:**
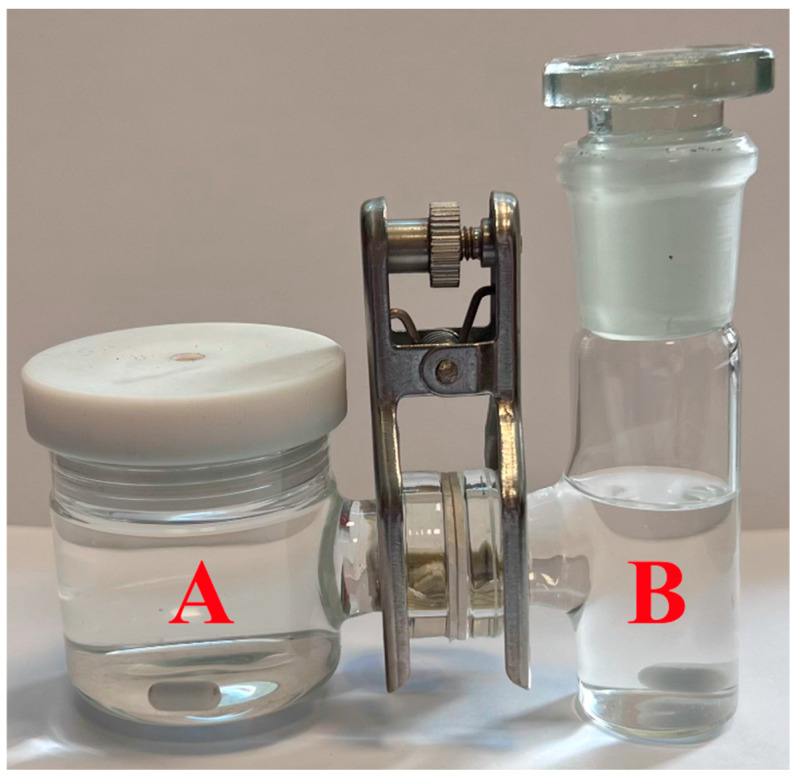
Photograph of the setup for permeation studies of 1-phenylethanol and mandelic acid through membranes based on the synthesized metathesis copolymers.

**General procedure 4.** Permeation of organic compounds through membranes based on the synthesized metathesis copolymers was studied using a setup composed of two vessels, «A» and «B», and a membrane with an effective (working) surface area of 2.0 cm^2^ sandwiched between two *O*-shaped silicon gaskets used to ensure the tightness of the entire system (Figure 1). The working volume of vessel «A» was 50 mL and that of vessel «B» was 35 mL. The permeation measurements were carried out at room temperature.

A total of 100 mL of a solution of benzoic acid (0.002 M) in hexane (“for HPLC” grade, TD Chimmed, Moscow, Russia, Product No. 00000007070) or methanol (“for HPLC” grade, TD Chimmed, Moscow, Russia, Product No. 00000005436) was prepared and equal amounts of the solution (50 mL) were poured into two vials denoted as “vial-1” and “vial-2”. To the solution in vial-1, 1-phenylethanol (or mandelic acid) was added until a concentration of 0.01 M and the combined solution was poured into vessel “A”. The solution in vial-2 was poured into vessel “B”. The solutions in the vessels “A” and “B” were stirred using magnetic stir bars at room temperature. Samples (0.8 mL) from vessel “B” were taken at certain intervals and analyzed via gas-liquid chromatography using a preliminarily plotted calibration curve relating signals of benzoic acid to those of 1-phenylethanol.

## 3. Results and Discussion

### 3.1. Synthesis of Metathesis Homopolymers

**Scheme 1:**
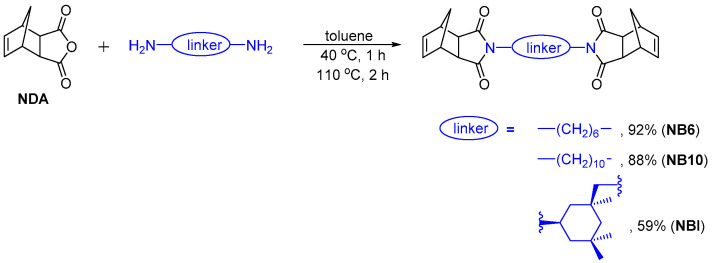
The synthesis of nadimides **NB6**, **NB10**, and **NBI**.

**Figure 2:**
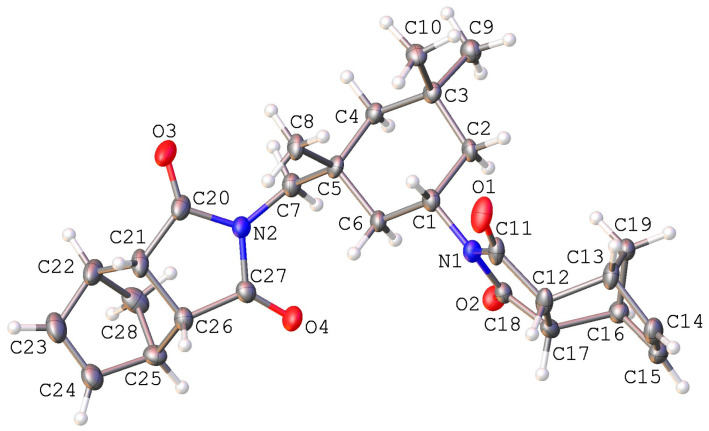
General view of the NBI molecule according to X-ray analysis. Atoms are represented by thermal ellipsoids (*p* = 50%).

To obtain cross-linked polymers via ROMP, in this work we used bifunctional monomers representing norbornene derivatives bearing two norbornene moieties, which can be involved in the polymerization process independently. The norbornene-type bifunctional monomers were bis(nadimides) based on hexamethylenediamine (**NB6**), decamethylenediamine (**NB10**), and 1*R*,3*S*-isophoronediamine (**NBI**, Scheme 1). These diamines are commercially available and widely used in the synthesis of various polyamides, polyurethanes, and polyimides. Two nadimides, **NB6** and **NB10**, were synthesized from *cis*-5-norbornene-*exo*-2,3-dicarboxylic anhydride (**NDA**, Scheme 1) following published procedures [47,48]. The reactions were carried out in toluene, and the products were purified via recrystallization from methanol or a methanol-chloroform mixture. The third nadimide, **NBI**, was synthesized under similar conditions using 1*R*,3*S*-isophoronediamine and isolated in pure form as a white solid with high T_m_. This bifunctional monomer was characterized via ^1^H and ^13^C NMR spectroscopy and mass spectrometry. The structure and absolute configuration of stereocenters in the **NBI** molecule were also confirmed via X-ray analysis (Figure 2).

**Scheme 2:**
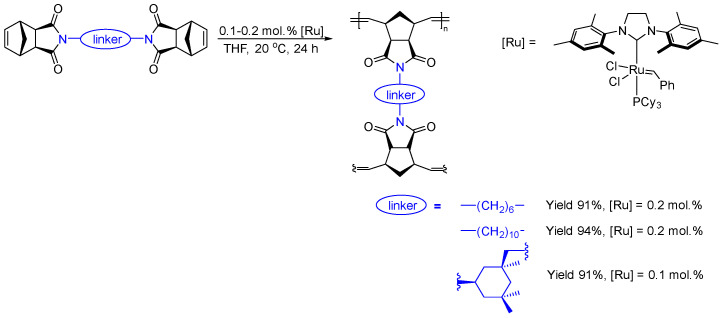
Metathesis homopolymerization of bifunctional nadimides **NB6**, **NB10**, and **NBI**.

**Figure 3:**
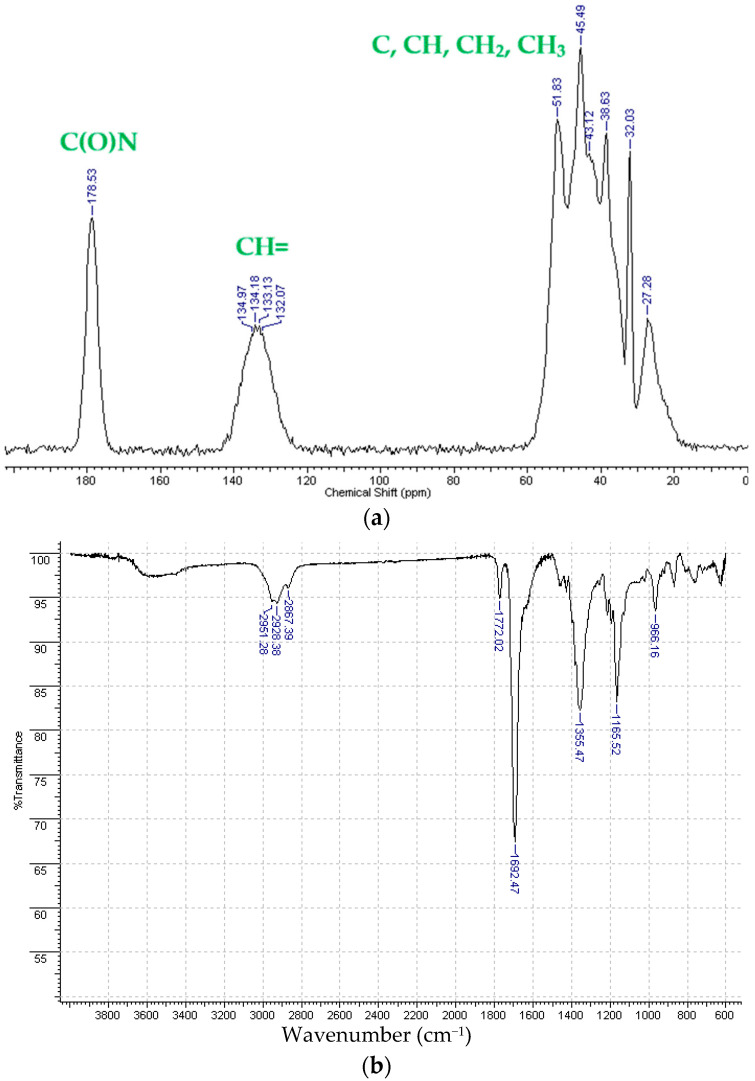
The CP/MAS ^13^C NMR spectrum (**a**) and IR spectrum (**b**) (ATR) of the metathesis homopolymer from **NBI**.

The metathesis homopolymerization of the synthesized bifunctional nadimides (**NB6**, **NB10**, and **NBI**) was carried out in the presence of the second-generation Grubbs catalyst (Scheme 2). Since the norbornene skeleton has an *exo*-configuration and the substituents are distant from the double bond being polymerized, the bifunctional monomers readily entered the metathesis polymerization reaction, which afforded insoluble polymers in nearly quantitative yields. The structure and purity of the synthesized polymers were confirmed via IR spectroscopy and solid-state ^13^C NMR spectroscopy (Figure 3 and Figures S1–S4). For example, in the solid-state ^13^C NMR spectra of the synthesized metathesis polymers, the signals of the carbon atoms of the C(N)=O moiety are clearly observed at about 178–180 ppm, while the carbon atoms of the C(H)=C(H) fragments are in the range of 120–140 ppm, appearing in place of the carbon atoms of the C(H)=C(H) fragment of the starting monomers (Figure 3a). Based on the NMR spectra, it is difficult to assess whether all of the norbornene fragments of the monomer have been completely involved in the metathesis polymerization because the double bond signals of the monomer and polymer overlap in the double bond region (120–140 ppm, Figure 3a). At the same time, in the IR spectra, the band at 1570 cm^−1^ (Figure 3b and Figures S1–S4), corresponding to the norbornene double bond, disappeared after the polymerization, which can evidence that almost all the norbornene fragments of the monomer were involved in the metathesis polymerization.

**Scheme 3:**
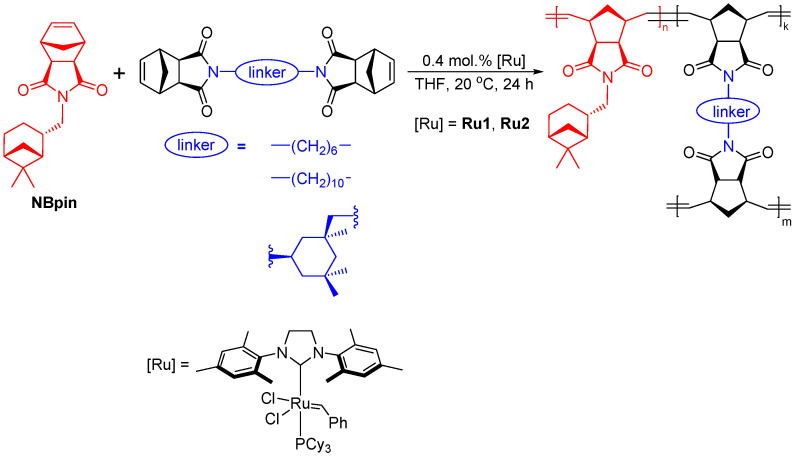
The metathesis copolymerization of bifunctional nadimides **NB6**, **NB10**, and **NBI** and monofunctional norbornene **NBpin**.

In spite of the high reactivity of the **NB6**, **NB10**, and **NBI** monomers in the polymerization reaction, we failed to fabricate thin polymer films based on them via solution casting of the reaction mixtures. However, the metathesis copolymerization of these bifunctional nadimides and a monofunctional norbornene derivative, which also contained the imido group, made it possible to obtain thin films based on cross-linked polymers (Scheme 3). A related norbornenedicarboxyimide bearing a pinanyl substituent (**NBpin**) was used as the main comonomer. The metathesis homopolymerization and properties of the homopolymer of **NBpin** were studied in our previous work [41].

The metathesis *co*polymerizations of **NBpin** and bifunctional nadimides were carried out in THF in the presence of the second-generation Grubbs catalyst (Scheme 3). Using the highly active catalyst and having optimized the reaction conditions, a quantitative conversion of the monomers was achieved at a reasonably low catalyst loading (0.4 mol.%). Indeed, no traces of unreacted monomers were detected after storage of the synthesized cross-linked polymers in organic solvents. The results obtained demonstrate that efficient copolymerization of **NBpin** and all three synthesized bifunctional nadimides is possible. Mention should be made that the content of the bifunctional monomer in our experiments varied from 5 to 90 mol%.

**Figure 4:**
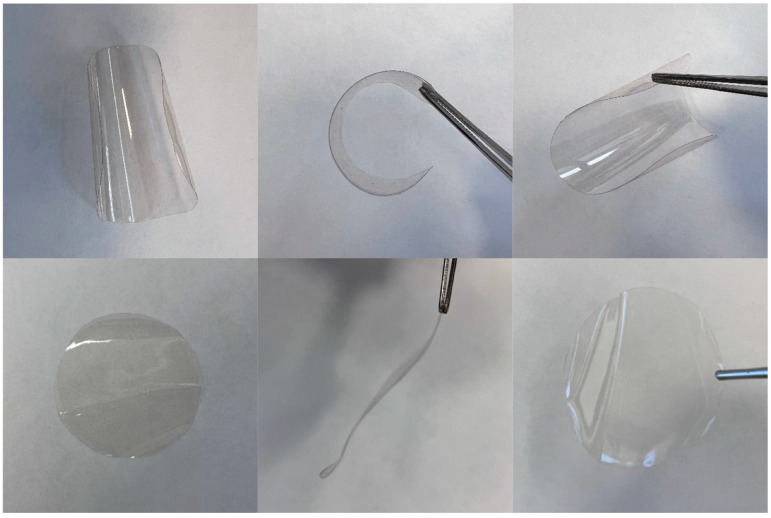
Photographs of a polymer film based on poly(**NBpin**-*co*-**NBI**) (90:10 mol.%) before (**upper** row) and after treatment with MeOH (**bottom** row).

**Figure 5:**
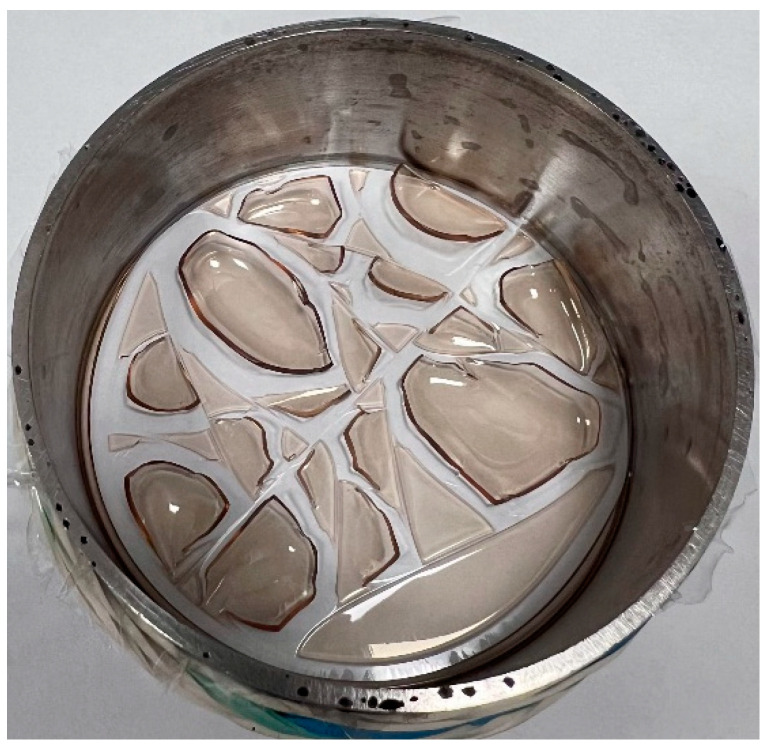
Photograph of a solution-cast polymer film based on poly(**NBpin**-*co*-**NBI**) (40:60 mol.%).

Then, we succeeded in fabricating transparent thin films based on the three cross-linked copolymers of **NBpin** and each bifunctional cross-linking agent via solution casting. The films’ pre- and post-methanol treatment images are shown in Figure 4. The films are insoluble and highly stable, do not swell in many organic solvents (Table 1), and have good mechanical properties (Table 2). In particular, reversible bending of the films is possible. It is noteworthy that if the content of the cross-linking agent in the comonomer mixtures exceeded 20 mol.%, the polymer films were usually brittle and spontaneously cracked upon solvent evaporation from the reaction mixture (Figure 5).

### 3.2. Physicochemical Properties of the Synthesized Metathesis Homo- and Copolymers

**Figure 6:**
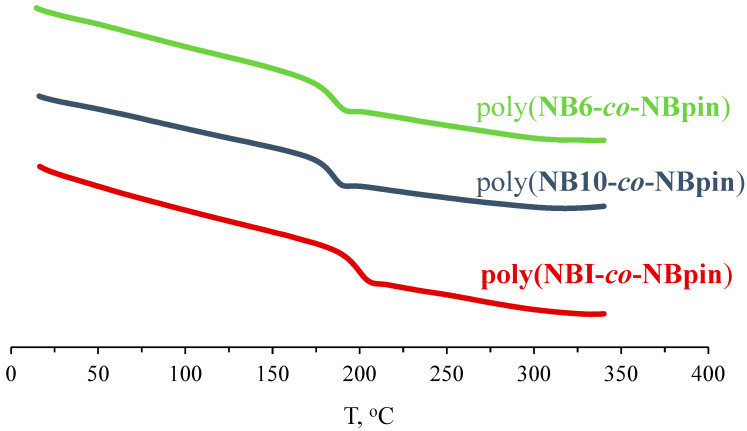
DSC curves of the synthesized metathesis copolymers of **NBpin** (90 mol.%) and bifunctional monomers (10 mol.%).

According to DSC data, all homo- and copolymers we have synthesized are glassy. The glass transition temperatures of the homopolymers of **NBI**, **NB6**, and **NB10** are above 270 °C and decrease as the linker elongates (Table 2). The glass transition temperatures of the copolymers of **NBpin** and bifunctional comonomers depend on the nature of the cross-linking agent. For instance, consider the copolymers containing **NBpin** (90 mol.%) and 10 mol.% of the cross-linking agent. As shown in Figure 6, their glass transition temperatures drop from 197 °C for poly(**NBpin**-*co*-**NBI**) to 186 °C for poly(**NBpin**-*co*-**NB10**). Interestingly, the glass transition temperature of the metathesis homopolymer based on **NBpin** (poly(**NBpin**)) is 188 °C [41], i.e., the introduction of the cross-linking agent has no effect or only slightly increases the glass transition temperature of the synthesized cross-linked polymer, which is determined by the flexibility and length of the linker in the bifunctional comonomer.

The DSC curves of the homopolymers of **NBI**, **NB6**, and **NB10** and those of the copolymers of these comonomers and **NBpin** of different compositions show no melting transitions (Figure 6). It follows that the synthesized cross-linked polymers are non-crystalline and materials fabricated from them are amorphous.

**Figure 7:**
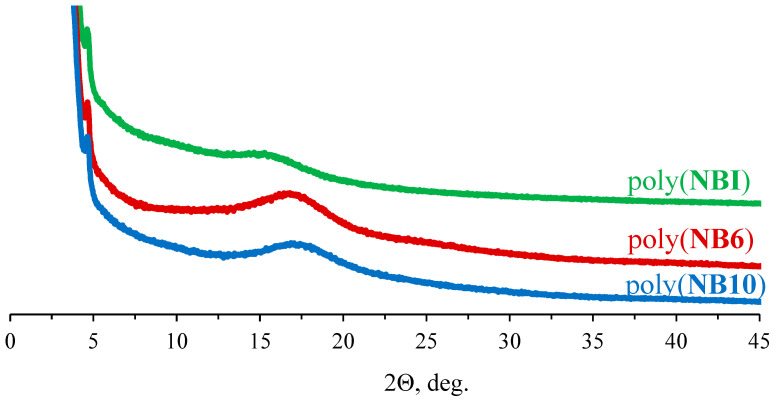
Powder XRD patterns of the synthesized metathesis homopolymers of **NB6**, **NB10**, and **NBI**.

The amorphous nature of the metathesis homo- and copolymers under study was additionally confirmed by powder XRD (Figure 7, Table 3). The corresponding powder XRD patterns exhibit single broad peaks. Peaks in the powder XRD patterns of the homopolymers of **NB10**, **NB6**, and **NBI** are shifted toward smaller 2θ values, thus indicating an increase in corresponding d-spacings that correlate with the inter-chain distances in the polymer. For instance, the d-spacing is 5.1 Å for poly(**NB10**), 5.3 Å for poly(**NB6**), and 5.8 Å for poly(**NBI**). Thus, the longer and more flexible the linker, the closer the packing of polymer chains.

**Figure 8:**
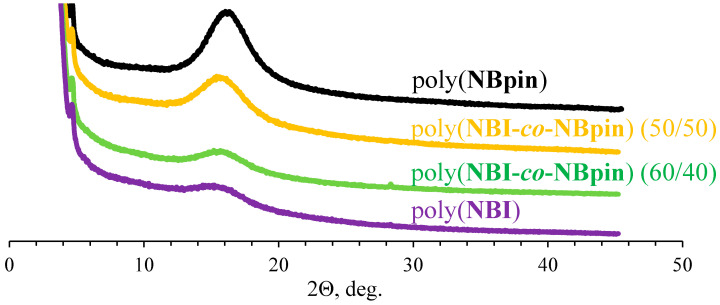
Powder XRD patterns of the synthesized metathesis copolymers of **NBpin** and bifunctional comonomer (**NBI**) of different composition.

Peaks in the powder XRD patterns of the copolymers of the bifunctional monomers under study and the monofunctional comonomer **NBpin** are observed between the 2θ values for the peaks of the corresponding homopolymers (poly(**NBpin**) and the homopolymer of the bifunctional monomer (see Figure 8 and Table 4)). The peak position is determined by the composition of the copolymer and is shifted to that homopolymer whose content in the copolymer increases. In turn, large d-spacings may indicate a looser packing of polymer chains. However, this is not true in all cases, even for the same series of structurally similar polymers.

**Figure 9:**
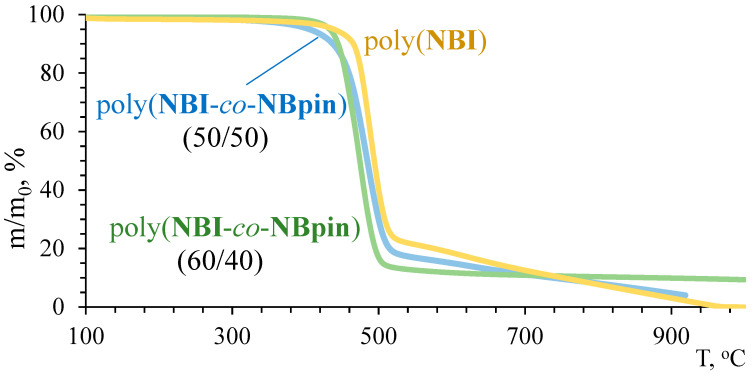
TGA curves of the synthesized metathesis homo- and copolymers based on **NBI** in an argon atmosphere.

**Figure 10:**
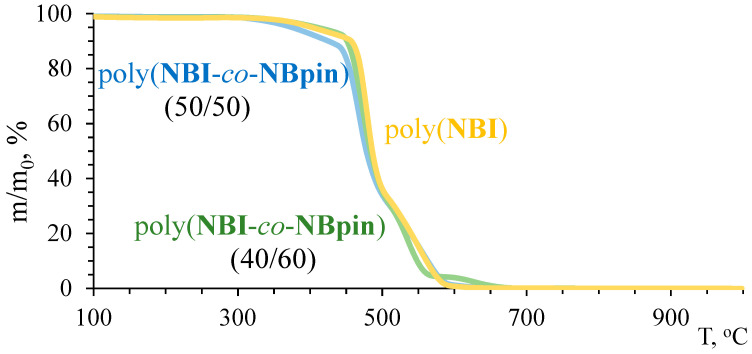
TGA curves of the synthesized metathesis homo- and copolymers based on **NBI** in air.

The synthesized cross-linked metathesis polymers demonstrated unexpectedly high thermal stability (Figure 9 and Figure 10, Table 2). Indeed, the temperatures corresponding to a sample weight loss of 5%, determined via TGA in an inert atmosphere, exceed 400 °C for all copolymers of **NBpin** and bifunctional comonomers. The homopolymer of **NBpin** has a similar decomposition temperature of 405 °C in an argon atmosphere and in air [41]. In all cases, decomposition of the polymers occurred in almost one step, and the residue after raising the TGA temperature to 900–1000 °C was less than 5%, thus indicating the formation of volatile products during the thermolysis. One can assume that both the main and side (co)polymer chains break down at temperatures close to 400 °C.

### 3.3. Permeability Study of the Synthesized Cross-Linked Copolymers

**Figure 11:**
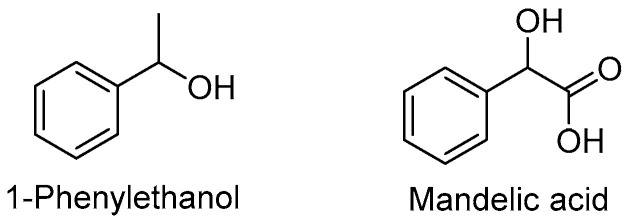
Structural formulas of 1-phenylethanol and mandelic acid, which were used to evaluate the permeability of membranes based on the synthesized metathesis polymers.

Permeation of organic compounds through the fabricated membranes was studied using the setup shown in Figure 1 in three media, viz., methanol, hexane, and an acetone:methanol mixture (4:1 *v*/*v*), taking 1-phenylethanol and mandelic acid (Figure 11) as examples. Molecules of the test compounds contain a hydroxyl and a carboxyl functional group, respectively. The choice of these molecules is due to their availability, the presence of functional groups present in many pharmaceutically active compounds, and the possibility to evaluate the effect of the presence of functional groups of a different nature on permeability. The effective surface area of the membrane was ca. 2.0 cm^2^, and the membrane thickness was mostly in the range of 77–97 µm. The concentration of the racemic organic compound in the feed solution was 0.01 M.

**Figure 12:**
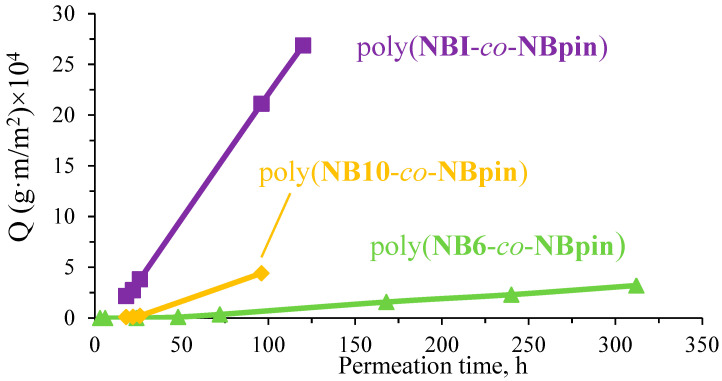
Normalized amount (*Q*) of permeated 1-phenylethanol plotted vs. permeation time through the studied copolymers in hexane (25 °C, the concentration of 1-phenylethanol in the feed solution was 0.01 M).

**Figure 13:**
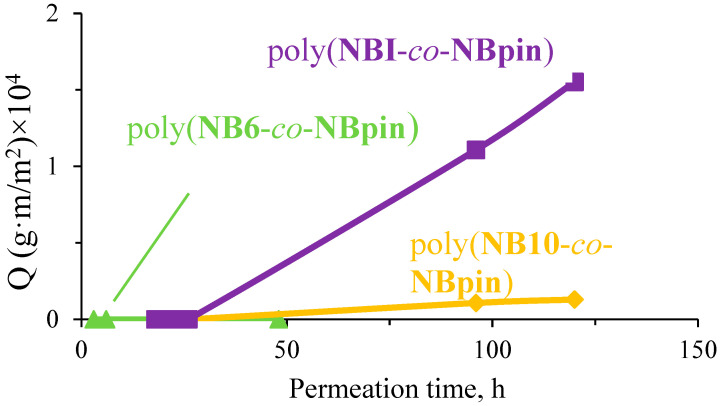
Normalized amount (*Q*) of permeated 1-phenylethanol plotted vs. permeation time through the studied copolymers in methanol (25 °C, the concentration of 1-phenylethanol in the feed solution was 0.01 M).

The cross-linking agent, penetrant, and medium (solvent) all had a significant impact on the membranes under study’s permeability. For example, the 1-phenylethanol permeation flux through the films based on the cross-linked metathesis polynorbornenes in hexane was more than tenfold higher than in methanol (Figure 12 and Figure 13). The reason for the significant permeability difference between the films made from similar polymeric materials in two different media can be attributed to the polymers’ increased degree of swelling in hexane, which increases the inter-chain lengths and increases polymer chain mobility.

The nature of the cross-linking agent is the next factor that has a strong effect on the permeability of the cross-linked metathesis polynorbornenes. A comparison of the permeation fluxes of 1-phenylethanol through the films under study demonstrated that the longer the spacer in the cross-linking agent, the more permeable the synthesized cross-linked copolymers (Figure 12 and Figure 13). A possible explanation is as follows: the longer the linker, the higher the mobility of the main polymer chains (this is indirectly confirmed by DSC data) and thus the lower the energy barrier to diffusion of the penetrant molecules. The highest permeability was observed for the copolymers of **NBpin** and **NBI** (Figure 12). The permeation flux of 1-phenylethanol through poly(**NBpin**-*co*-**NBI**) in hexane was nearly five times higher than through the film based on poly(**NBpin**-*co*-**NB10**). This is most likely due to the more rigid structure of the cyclic spacer compared to the polymethylene one. Like the long length, the high rigidity of the spacer provides longer inter-chain distances, and probably, the more branched structure of the hydrocarbon linker additionally favors a higher degree of swelling of the copolymer in the related hydrocarbon solvent, hexane.

**Figure 14:**
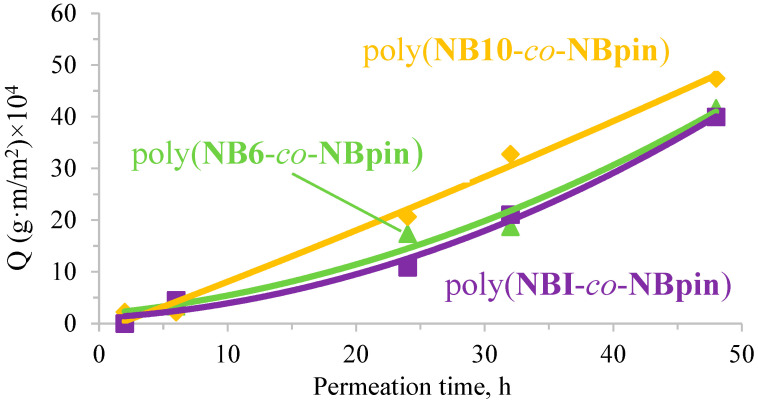
Normalized amount (Q) of permeated mandelic acid plotted vs. permeation time through the studied copolymers in the acetone:methanol mixture (4:1 *v*/*v*; 25 °C, the concentration of mandelic acid in the feed solution was 0.01 M).

The permeation of mandelic acid through the films based on the synthesized copolymers in methanol and hexane was very low, being much lower than that of 1-phenylethanol under similar conditions. However, the use of the acetone–methanol mixture instead of the other two solvents significantly changed the situation. The copolymers’ degree of swelling rose, which allowed for a significant improvement in mandelic acid permeability (Figure 14). Owing to the higher degree of swelling, the permeation flux of mandelic acid through the copolymers in question in the acetone–methanol mixture appeared to be even higher than that of 1-phenylethanol in hexane under similar conditions. Interestingly, in this case, the nature of the linker had little effect, and the permeation values of mandelic acid through the copolymers based on different bifunctional comonomers were close to one another. This behavior is most likely due to the comparable degrees of swelling of the tested copolymers in the presence of acetone. In turn, the observed higher permeability of 1-phenylethanol compared to that of mandelic acid is probably explained by the presence of two functional groups in mandelic acid, which enhance the interaction with the polymer matrix, and the larger size of mandelic acid molecules. Thus, the permeation flux of organic compounds through membranes based on the synthesized copolymers can be controlled by varying the nature of both the cross-linking agent and the solvent used.

## 4. Conclusions

The metathesis homo- and copolymerization of bifunctional nadimides based on diamines of different natures was studied systematically. The synthesized monomers bearing two reactive norbornene moieties are highly active in both metathesis homopolymerization and metathesis copolymerization with the related nadimide containing the β-pinene fragment in the presence of the second-generation Grubbs catalyst, which affords insoluble cross-linked polymers. All the synthesized polymers represent glassy, amorphous substances. The glass transition temperatures of the polymers can be controlled by varying the length of the spacer in the bifunctional monomer. The metathesis copolymerization conditions and the composition of the copolymers were optimized to ensure the fabrication of mechanically strong polymer films that are stable in various organic solvents. It was found that the permeability of membranes based on the synthesized copolymers to 1-phenylethanol and mandelic acid depends strongly on the nature of the cross-linking agent, penetrant, and solvent. The maximal permeability can be obtained using the bifunctional monomer with the long flexible spacer or with the shorter rigid spacer in the hydrocarbon solvent (hexane). The findings of the film permeability measurements and the methods for creating solution-cast polymer films described in this study present opportunities for further thorough research on the use of membranes based on the title polymers for the separation of organic component mixtures.

## Figures and Tables

**Table 1:** Stability of films based on cross-linked metathesis copolymers of **NBpin** (90 mol.%) and bifunctional comonomers (10 mol.%) in different organic solvents.

Bifunctional Comonomer	CH_3_CN	MeOH	Acetone	CH_2_Cl_2_	CHCl_3_	Toluene	Et_2_O	n-Hexane
**NBI**	±	+	±	−	−	−	+	+
**NB6**	±	+	±	−	−	−	+	+
**NB10**	±	+	±	−	−	−	+	+

“+”—polymer film did not change; “±”—polymer film became more flexible (like a rubber, e.g., see Figure S5); “−”—polymer film significantly swelled or was destroyed.

**Table 2:** Young’s modulus, tensile strength, and elongation at break values of the synthesized cross-linked metathesis copolymers based on **NBpin** (90 mol.%) and bifunctional comonomers (10 mol.%).

Bifunctional Comonomer	*T*_g_, °C ^a^	*T*_d5%_, °C ^b^	*d*, g cm^−3^	Tensile Strength, σ, MPa	Elongation at Break, ε, %	Young’s Modulus, *E*, MPa
**NBI**	>350	406	1.147	20	14	960
**NB6**	>350	403	1.140	33	16	-
**NB10**	272	414	1.135	29	12	1170

^a^ Glass transition temperature of *homo*polymers from bifunctional monomers, as determined via DSC. ^b^ Decomposition temperature at which the sample weight loss is 5% (according to TGA data obtained in argon atmosphere).

**Table 3:** Powder XRD data for the metathesis homopolymers of **NBI**, **NB6**, **NB10**, unsubstituted norbornene (poly(NB)), and **NBpin**.

Parameter	Homopolymer				
	poly(NBI)	poly(NB6)	poly(NB10)	poly(NBpin) [41]	poly(NB) [49]
2θ_1_, °	15.3	16.9	17.5	16.2	18.1
d_1_, Å	5.8	5.3	5.1	5.4	4.9

**Table 4:** Powder XRD data for the metathesis copolymers of **NBI** and **NBpin** and for the metathesis homopolymers of **NBI** and **NBpin**.

Parameter	Polymer				
	poly(NBpin) [41]	poly(NBI-*co*-NBpin) (10/90) ^a^	poly(NBI-*co*-NBpin) (50/50) ^a^	poly(NBI-*co*-NBpin) (60/40) ^a^	poly(NBI)
2θ_1_, °	15.3	16.9	17.5	16.2	18.1
d_1_, Å	5.8	5.3	5.1	5.4	4.9

^a^ Copolymer composition, **NBI**:**NBpin** (mol. %).

## Data Availability

The results of X-ray analysis of NBI are available free of charge as a file of Supplementary Materials, further inquiries can be directed to the corresponding author.

## Supplementary Materials

The following supporting information can be downloaded at: https://www.mdpi.com/article/10.3390/polym16182671/s1, Table S1: Crystal data and structure refinement details for **NBI**. Figure S1. IR spectra (ATR) of **NB6** and the metathesis homopolymer from **NB6**. Figure S2. IR spectra (ATR) of **NB10** and the metathesis homopolymer from **NB10**. Figure S3. IR spectra (ATR) of **NBI** and the metathesis homopolymer from **NBI**. Figure S4. IR spectra (ATR) of **NBpin** and the metathesis comopolymer from **NBI** and **NBpin**. Figure S5. A photograph of a polymer film based on poly(**NBpin**-*co*-**NBI**) (90:10 mol.%) after the emersion in acetone for 24 h (the film was stretched along the opposite corners using tweezers. The film stretches but does not break down).
