# Supplementary material for: Cross-Linked Metathesis Polynorbornenes Based on Nadimides Bearing Hydrocarbon Substituents: Synthesis and Physicochemical Properties
Source: Polymers (Basel). 2024 Sep 22;16(18):2671. doi: 10.3390/polym16182671 (PMC11436105; doi:10.3390/polym16182671)
Supplement: Supplementary file 1 [file polymers-16-02671-s001.zip › polymers-3210777-supplementary.pdf]

Supplementary Material

Cross-linked metathesis polynorbornenes based on nadimides bearing hydrocarbon substituents: synthesis and physicochemical properties

Kirill S. Sadovnikov,[1] Ivan V. Nazarov,[1] Vsevolod A. Zhigarev,[1] Anastasia A. Danshina,[2,3] Igor S. Makarov,[1] and Maxim V. Bermeshev[1]*

[1]A. V. Topchiev Institute of Petrochemical Synthesis, Russian Academy of Sciences, 29 Leninsky prospekt, 119991 Moscow, Russian Federation.

[2]A.N. Nesmeyanov Institute of Organoelement Compounds, Russian Academy of Sciences, 28 Vavilova str., Moscow, 119334, Russian Federation.

[3]Moscow Center for Advanced Studies, 20, Kulakova Str., Moscow, Russia.

*E-mail: bmv@ips.ac.ru; Tel.: +7 495 647 59 27*379

Table of contents

X-ray diffraction data for NBI 2

IR spectra of the synthesized monomers and polymers 3

The photo of polymer films 7

X-ray diffraction data for NBI

Table S1. Crystal data and structure refinement details for **NBI**.

	**NBI**
Empirical formula	$C_{28}H_{34}N_2O_4$
Formula weight	462.57
T, K	120
Crystal system	Orthorhombic
Space group	$P2_12_12_1$
Z	4
a, Å	7.0666(12)
b, Å	10.2915(17)
c, Å	32.255(5)
α, °	90
β, °	90
γ, °	90
V, Å^3	2345.7(7)
D_{calc} (g cm^{-1})	1.310
μ, cm^{-1}	0.87
F(000)	992
$2\theta_{max}$, °	58
Reflections measured	27656
Independent reflections	6207
Observed reflections [I > 2σ(I)]	4751
Parameters	311
R1	0.0634
wR2	0.1338
GOF	1.049
$\Delta\rho_{max}$/ $\Delta\rho_{min}$ (e Å^{-3})	0.279/-0.236

IR spectra of the synthesized monomers and polymers

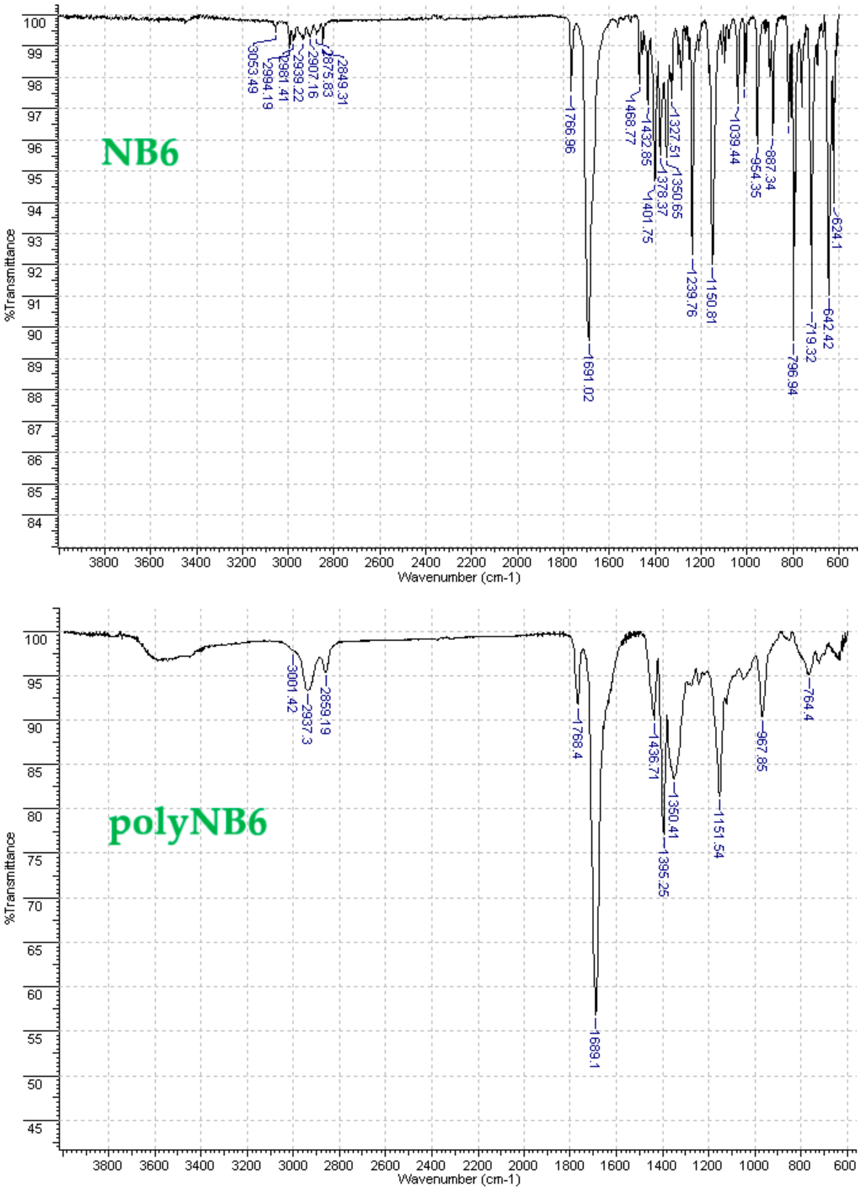

Figure S1. IR spectra (ATR) of **NB6** and the metathesis homopolymer from **NB6**.

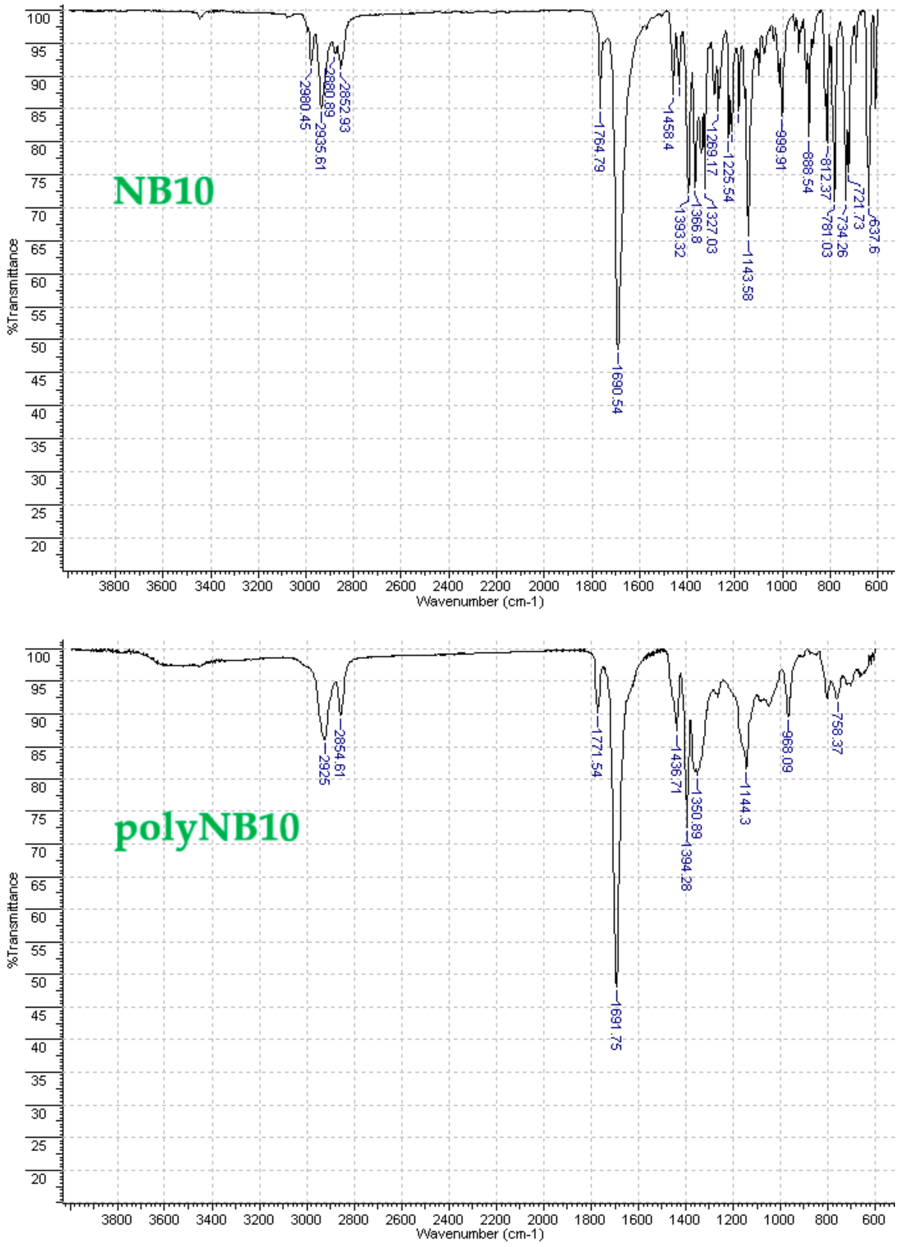

Figure S2. IR spectra (ATR) of **NB10** and the metathesis homopolymer from **NB10**.

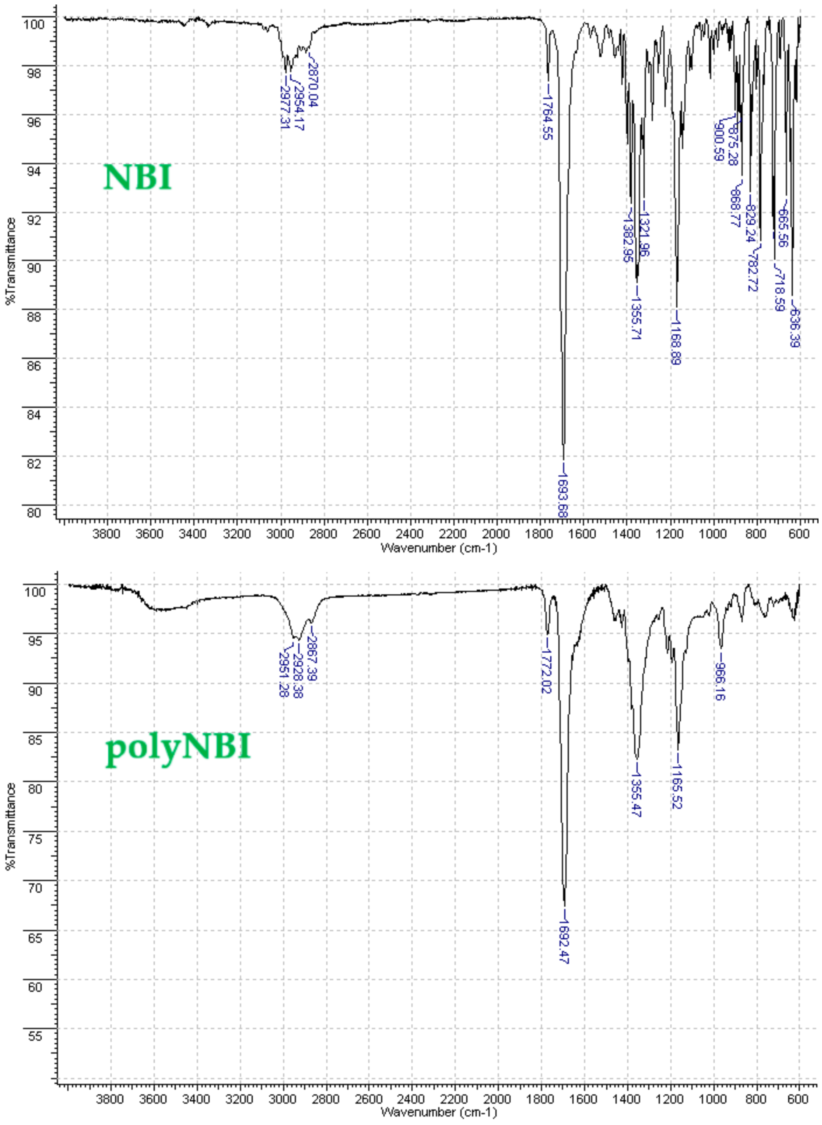

Figure S3. IR spectra (ATR) of **NBI** and the metathesis homopolymer from **NBI**.

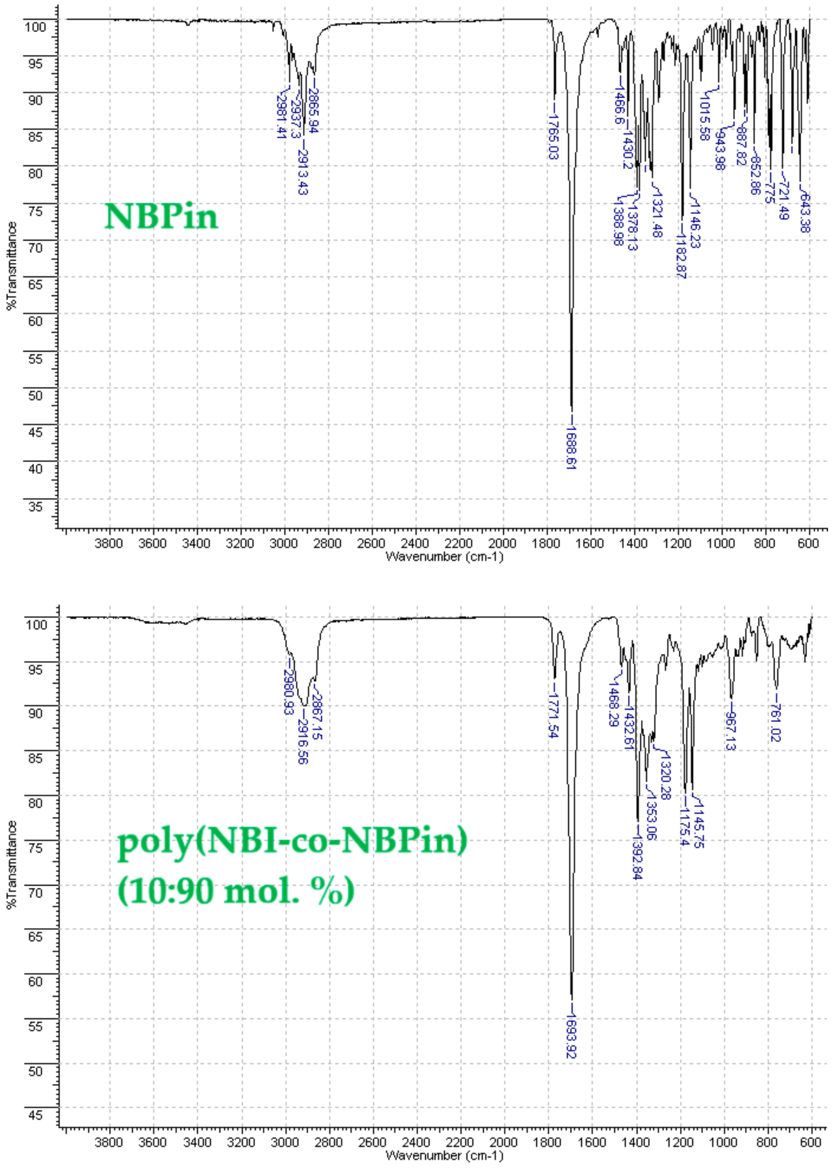

Figure S4. IR spectra (ATR) of **NBpin** and the metathesis comopolymer from **NBI** and **NBpin**.

The photo of polymer films

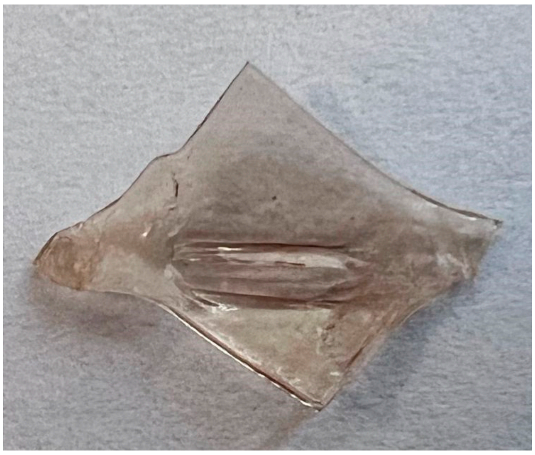

Figure S5. A photograph of a polymer film based on poly(**NBpin**-*co*-**NBI**) (90 : 10 mol.%) after the emersion in acetone for 24 h (the film was stretched along the opposite corners using tweezers. The film stretches but does not break down).
